# Patchouli Alcohol as a Potential Anti-Cariogenic Compound Targeting *Streptococcus mutans*

**DOI:** 10.3390/molecules31152577

**Published:** 2026-07-24

**Authors:** Qianying Chen, Chong Feng, Zhimin Zhao, Depo Yang, Wenzhe Yang

**Affiliations:** 1 School of Pharmaceutical Sciences, Sun Yat-sen University, Guangzhou 510006, China; chenqy295@mail.sysu.edu.cn (Q.C.); fengch28@mail2.sysu.edu.cn (C.F.);; 2 Accuramed Technology (Guangzhou) Limited, Guangzhou 510700, China

**Keywords:** patchouli alcohol, *Streptococcus mutans*, virulence factors, dental caries, biofilm, antibacterial activity

## Abstract

Patchouli alcohol, a natural tricyclic sesquiterpene isolated from *Pogostemon cablin*, exhibits promising antimicrobial properties, yet its activity against *Streptococcus mutans* (*S. mutans*), a major cariogenic bacterium associated with dental caries, has not been characterized. This study determined the minimum inhibitory concentration (MIC) and minimum bactericidal concentration (MBC) of patchouli alcohol against *S. mutans* to be 40 and 80 μg/mL, respectively. A series of phenotypic assays showed that patchouli alcohol delayed bacterial growth and culture acidification, reduced extracellular polysaccharide production, and decreased the biomass and metabolic activity of established *S. mutans* biofilms. Scanning electron microscopy showed treatment-associated changes in the surface morphology of *S. mutans*, with more evident deformation at MIC and 2MIC. Real-time quantitative PCR showed that sub-MIC treatment (20 μg/mL) significantly downregulated virulence genes associated with adhesion (*gbpB* and *spaP*), EPS synthesis (*gtfB*, *gtfC*, *gtfD*, and *ftf*), acid production (*ldh*, *atpF*, and *atpD*), and the two-component regulatory and quorum sensing systems (*vicK*, *vicR*, and *luxS*). These results suggest that patchouli alcohol may serve as a potential natural anti-cariogenic candidate targeting *S. mutans*-associated virulence traits under in vitro conditions.

## 1. Introduction

Dental caries is a multifactorial, biofilm-mediated disease affecting the hard tissues of susceptible teeth. It is among the most prevalent noncommunicable diseases worldwide and remains a major public-health burden [1]. Dental caries is now recognized as a multifactorial disease driven by ecological dysbiosis within acidogenic and aciduric multispecies biofilms, leading to sustained acid production, mineral loss, and structural damage of the teeth [2]. Caries development is closely associated with disruption of the oral microbial balance. Among cariogenic bacteria, *S. mutans* has long been regarded as an important contributor to dental caries because of its strong acidogenicity, aciduricity, and biofilm-forming capacity [3]. Although dental caries involves complex microbial communities, *S. mutans* remains a widely used model organism for studying cariogenic virulence because of its roles in adhesion, acid production, acid tolerance, and extracellular polysaccharide synthesis [4]. *S. mutans* utilizes carbohydrates such as sucrose and glucans to form biofilms on the surfaces of teeth and soft tissues, which results in the production of organic acids that lead to enamel demineralization. In the presence of sucrose, *S. mutans* produces extracellular polysaccharides that contribute to the formation of a biofilm matrix, thereby promoting bacterial adhesion and enamel demineralization. Therefore, targeting *S. mutans*-associated virulence traits, such as adhesion, acid production, acid tolerance, EPS synthesis, and biofilm development, remains a useful strategy for investigating anti-cariogenic candidates in vitro [5]. Conventional treatments for dental caries involve antimicrobial therapies (e.g., chlorhexidine, fluoride) aimed at eliminating cariogenic bacteria in the oral cavity. However, the overuse of these antimicrobial agents has disrupted the homeostasis of oral microecology [6]. Therefore, safer adjunctive strategies for controlling cariogenic bacteria and their virulence traits are still needed.

Plant-derived compounds have been explored as potential adjunctive agents for controlling cariogenic bacteria because of their structural diversity and biological activity. Several plant-derived compounds have been reported to inhibit *S. mutans* growth or attenuate cariogenic phenotypes in vitro [7]. Ke et al. [8] analyzed the effects of curcumin on *S. mutans* through transcriptomic methodologies, revealing that curcumin may suppress biofilm activity by regulating the expression of genes associated with biofilm formation, thereby significantly inhibiting both biofilm formation and degradation. Aragao et al. [9] investigated the inhibitory effects of epigallocatechin gallate (EGCG) from green tea on bacterial growth, acid production, and sugar uptake via the phosphoenolpyruvate-dependent phosphotransferase system (PEP-PTS), suggesting that EGCG may exert short-term antibacterial effects against dental caries. Feng et al. [10] focused on cranberries, employing multi-step separation and structural determination techniques to isolate oligomers of cranberry type A proanthocyanidins (PACs) and study their impact on biofilm three-dimensional structure and transcriptomics. The experimental results demonstrated that PACs possess the capability to inhibit bacterial adhesion and infection arising from biofilms.

Patchouli alcohol is a natural tricyclic sesquiterpene with various promising medicinal activities reported, including anti-influenza virus, antidepressant, vasodilatory, lung-protective, neuroprotective, anti-ulcer, anti-colitis, prebiotic, anti-inflammatory, anti-cancer, and protective effects against metabolic diseases [11]. Its antibacterial and antiviral properties against microorganisms are particularly noteworthy. Notably, patchouli alcohol can inhibit influenza A (H1N1) by directly interacting with virus particles and disrupting the entry of the virus into host cells through inhibition of the PI3K/Akt and ERK/MAPK signaling pathways, which are critical in viral invasion and the viral life cycle [12]. Previous studies have reported that patchouli alcohol and patchouli oil exhibit antibacterial activity against several Gram-positive and Gram-negative bacteria in vitro [13]. Additionally, it demonstrates a protective effect on gastric mucosa and acts selectively against *Helicobacter pylori* without disrupting other gastrointestinal flora, displaying a distinct mechanism of action compared to conventional antibacterial drugs, thus indicating superior safety [14]. We selected patchouli alcohol for further evaluation because it showed activity against *S. mutans*-associated phenotypes in our preliminary screening. Patchouli alcohol is a naturally occurring constituent of *Pogostemon cablin*, is commercially available, and has been reported to exhibit antimicrobial activity against several microorganisms. Therefore, this study evaluated the antibacterial and established-biofilm effects of patchouli alcohol against *S. mutans* and examined its effects on selected cariogenic virulence traits in a single-species in vitro model.

## 2. Materials and Methods

### 2.1. Medium and Reagents

Brain Heart Infusion Broth (BHI) was obtained from HuanKai Microbial (Guangzhou, China), and Reinforced Clostridium Agar (RCA) was obtained from Shanghai Acmec Biochemical Technology Co., Ltd. (Shanghai, China). Patchouli alcohol was purchased from Shanghai Yuanye Bio-Technology Co., Ltd. (Shanghai, China) and stored at 4 °C until use. The compound was obtained as a commercially available reference-grade product with a purity of >98% according to the supplier’s certificate of analysis. DMSO was purchased from Sigma-Aldrich (Burlington, MA, USA). The Annexin V-FITC/PI detection kit was purchased from Yeasen Biotechnology Co., Ltd. (Shanghai, China).

### 2.2. Bacterial Strains and Culture Conditions

The *S. mutans* strain ATCC 25175 was obtained from the Guangdong Microbial Strain Preservation Center. Frozen stocks were thawed and streaked onto RCA plates, followed by incubation at 37 °C for 24 h under anaerobic conditions. A single colony was then transferred into BHI broth and cultured at 37 °C with shaking at 180 rpm. The bacterial suspension was adjusted to OD600 = 1.0, corresponding to approximately 1 × 10^8^ CFU/mL, and diluted as required for subsequent experiments. The bacterial solution was adjusted to an optical density (OD600) of 1.0, corresponding to approximately 1 × 10^8^ CFU/mL, and subsequently diluted ten- to one-hundred-fold for further experiments.

### 2.3. Minimum Inhibitory Concentration (MIC) and Minimum Bactericidal Concentration (MBC)

The minimum inhibitory concentration (MIC) and minimum bactericidal concentration (MBC) were determined to evaluate the antimicrobial efficacy of patchouli alcohol. This experimental method was adapted from the literature [15], with slight modifications. The bacterial solution was adjusted to a concentration of 1 × 10^6^ CFU/mL. Concurrently, patchouli alcohol was dissolved in DMSO and diluted with BHI medium to concentrations ranging from 1.25 to 320 μg/mL using a double dilution method. Chlorhexidine served as the positive control, prepared at concentrations ranging from 0.15625 to 20 μg/mL using a double dilution method. A total of 100 µL of each prepared bacterial solution and corresponding pharmaceutical solution was added to a 96-well round-bottom plate. After thorough mixing, the bacteria were incubated at 37 °C for 24 h, with the lowest inhibitory concentration defined as the point at which no visible bacterial growth was observed. The contents of the wells at 100 µL MIC, 2MIC, and 4MIC concentrations were uniformly spread onto the plates, which were subsequently cultured for 24 h. The minimum bactericidal concentration (MBC) was defined as the lowest concentration that resulted in a 99.9% reduction in bacterial viability.

### 2.4. Growth Curve

Patchouli alcohol was diluted in BHI medium to the concentrations of MIC, 1/2MIC, 1/4MIC, and 1/8MIC. A total of 1 mL of each concentration was then added to separate test tubes, followed by the addition of 1 mL of bacterial solution with a concentration of 1 × 10^6^ CFU/mL. The negative control group was treated with corresponding concentrations of DMSO only. The assay was independently repeated three times. In each independent experiment, separate tubes were prepared for each treatment group and time point. At 0, 4, 8, 12, 24, and 48 h, one tube from each group was collected to determine the OD600 value and subsequently plot a growth curve. Sterile BHI medium containing patchouli alcohol at the same concentrations used in the bacterial growth assay, but without bacteria, was prepared and measured at OD600.

### 2.5. Acid Production Curve

Patchouli alcohol was diluted in BHI medium (containing 1% sucrose) to the concentrations of MIC, 1/2MIC, 1/4MIC, and 1/8MIC. A total of 4.5 mL of the prepared patchouli alcohol solution was added to a test tube, followed by 0.5 mL of a bacterial solution at 1 × 10^7^ CFU/mL. Corresponding concentrations of DMSO were used as the negative control. Six parallel replicas were established for each group and cultured in a shaking incubator at 37 °C under anaerobic conditions. One tube was removed from each group at the specified time points of 0, 4, 8, 12, 24, and 30 h. The pH value of the supernatant was measured, and an acid production curve was subsequently plotted.

### 2.6. Crystal Violet Staining for Established Biofilm Treatment Assay

Crystal violet staining was employed to assess the impact of patchouli alcohol on the established biofilm biomass of *S. mutans*. A total of 100 µL of BHI medium and 100 µL of a bacterial suspension of 1 × 10^6^ CFU/mL were added to each well of a 96-well flat-bottom cell culture plate. The plate was incubated anaerobically at 37 °C for 24 h to allow biofilm formation. Following incubation, the supernatant was carefully discarded, and the wells were washed gently twice with sterile PBS buffer before being allowed to air dry for 5 min. Next, 100 µL of varying concentrations of patchouli alcohol was added to each well. Established biofilms were then treated with patchouli alcohol at 4 MIC, 2 MIC, MIC, 1/2 MIC, or 1/4 MIC, while the negative control group was treated with the corresponding concentrations of DMSO. Five independent replicate wells were prepared for each treatment group in the 96-well biofilm assay. After a subsequent 24 h anaerobic culture at 37 °C, the supernatant was removed, and the wells were gently washed with sterile PBS buffer. The wells were then fixed with 100 µL of methanol for 10 min. Following the removal of methanol, the wells were air-dried for 5 min. Subsequently, 100 µL of a 0.1% crystal violet solution was added to stain the biofilm for 20 min, after which the solution was discarded. After washing away excess crystal violet with PBS, 200 µL of 95% ethanol was added to each well, which was then agitated for 15 min at room temperature. Finally, the absorbance was measured at 570 nm using a microplate reader (BioTek Synergy H1, Agilent, Santa Clara, CA, USA). The remaining established-biofilm biomass was calculated using the following formula:

Remaining biofilm biomass (%) = (Experimental group OD at 570 nm − Blank group OD at 570 nm)/ (Negative control group OD at 570 nm − Blank group OD at 570 nm) × 100%

### 2.7. MTT Assay

The methylthiazolyldiphenyl–tetrazolium bromide (MTT) assay aimed to evaluate the effect of patchouli alcohol on the metabolic activity of biofilm formed by *S. mutans* over 24 h. The biofilm was established according to the method described in Section 2.6. After incubation for 24 h, the supernatant was removed, and 100 µL of MTT solution (5 mg/mL) was added to each well. Following incubation at 37 °C in darkness for 2 h, the supernatant was carefully discarded, and 200 µL of DMSO was added to each well to dissolve the dye; the mixture was then shaken for 10 min at room temperature. The optical density (OD) value was determined at 490 nm using a microplate reader.

### 2.8. Scanning Electron Microscopy (SEM) Observation

Patchouli alcohol concentrations of 80, 40, 20, 10, and 5 μg/mL were prepared, corresponding to 2MIC, MIC, 1/2MIC, 1/4MIC, and 1/8MIC, respectively. The negative control group consisted of DMSO at corresponding concentrations. The bacteria were washed twice with PBS, after which 2.5% glutaraldehyde was added for fixation at 4 °C for 5 h. Following fixation, the bacteria were soaked and washed with PBS 3–5 times for 20 min each wash, followed by gradient dehydration using 30%, 50%, and 70% ethanol for 15 min each. The samples were then soaked in 70% ethanol overnight, followed by dehydration in a gradient of 90% and 100% ethanol for 15 min each. Finally, samples were exchanged with tert-butanol and soaked three times for 15 min each. Following freeze-drying, the samples were gold-coated in a vacuum environment, and bacterial morphology was observed using scanning electron microscopy.

### 2.9. Extracellular Polysaccharide (EPS) Quantification

Following the method outlined in Section 2.5, patchouli alcohol solutions with concentrations of 80, 40, 20, 10, and 5 μg/mL were prepared. The negative control group consisted of DMSO at the corresponding concentrations. The solutions were incubated anaerobically in a temperature-controlled incubator at 37 °C for 24 h, followed by centrifugation at 4500 rpm for 8 min. The supernatant was discarded, and the precipitate was washed twice with ultrapure water. After washing by centrifugation, the supernatant was collected, and three volumes of 95% ethanol were added to it at 4 °C and allowed to precipitate overnight. The precipitates collected represented the water-soluble extracellular polysaccharides (SEPS). The precipitate obtained after washing with ultrapure water in the previous step was then resuspended in 0.1 mol/L NaOH solution for further washing, after which it was centrifuged, and the supernatant was collected as water-insoluble exopolysaccharides (IEPS). The SEPS and IEPS fractions were quantified using the anthrone–sulfuric acid method.

### 2.10. Assessment of Membrane Integrity by Annexin V-FITC/PI Staining

An Annexin V-FITC/PI staining kit(Yeasen Biotechnology Co., Ltd., Shanghai, China) was used to assess changes in bacterial membrane integrity by flow cytometry. Bacteria in the logarithmic growth phase were collected, and the concentration of the bacterial solution was adjusted to 1 × 10^7^ CFU/mL, to which patchouli alcohol (1/2MIC, MIC, 2MIC, and 4MIC) was added. DMSO at the corresponding concentration served as the negative control group. The mixture was incubated at 37 °C and 180 rpm in a temperature-controlled incubator for 8 h, followed by centrifugation at 4500 rpm. The bacteria were rinsed three times with PBS buffer, and the bacterial suspension was diluted to a concentration of 1 × 10^6^ CFU/mL. The mixture was then centrifuged again and analyzed by flow cytometry according to the instructions of the Annexin V-FITC/PI detection kit. After discarding the PBS, 100 μL of 1× Binding Buffer was added to the resuspended cells, and 5 μL of Annexin V-FITC along with 5 μL of PI dye solution were added and mixed thoroughly. The reaction mixture was protected from light and incubated at room temperature for 15 min. Subsequently, another 400 μL of 1× Binding Buffer was added, mixed well, and the samples were placed on ice. The samples were analyzed by flow cytometry within 1 h of preparation. Given that Annexin V-FITC/PI staining was originally developed for eukaryotic apoptosis detection, the results were interpreted as changes in bacterial membrane integrity rather than evidence of apoptosis. Flow cytometric analysis was performed using a CytoFLEX flow cytometer (Beckman Coulter, Indianapolis, IN, USA). For each sample, at least 50,000 single-bacterial-cell events were acquired. When low-abundance-stained subpopulations were analyzed, no fewer than 100,000 single-bacterial-cell events were collected to reduce sampling error. Bacterial cells were first gated according to forward scatter (FSC) and side scatter (SSC) profiles to exclude debris, background noise, and bacterial aggregates, and only single bacterial cells were included for subsequent quantitative analysis. Unstained controls, Annexin V-FITC single-stained controls, and PI single-stained controls were used to establish quadrant gates and fluorescence compensation. The proportions of Annexin V-FITC-positive/PI-negative, Annexin V-FITC-positive/PI-positive, PI-positive, and double-negative cells were quantified using FlowJo v10 software. Three independent biological replicates were performed for each treatment group.

### 2.11. Real-Time Quantitative PCR Assay

Single colonies of *S. mutans* were selected for pure culture, and the cultured bacterial solution was diluted to a concentration of 1 × 10^6^ CFU for subsequent experiments. The medium utilized in this experiment was BHI medium supplemented with 10% sucrose. 1/2MIC (20 μg/mL) was selected to minimize confounding by extensive growth inhibition/cell death while allowing assessment of virulence-related transcriptional responses. A total of 700 μL of the bacterial solution and 700 μL of patchouli alcohol at a concentration of 20 μg/mL were added to a 1.5 mL EP tube, with corresponding concentrations of DMSO serving as the negative control group. Three replicate groups were established for each treatment condition. The bacterial cultures were incubated at 180 rpm and 37 °C under anaerobic conditions for 16 h, followed by centrifugation at 12,000 rpm for 2 min to collect the bacteria. The collected bacterial pellets were immediately frozen in liquid nitrogen for 15 min and subsequently stored in an ultra-low-temperature freezer.

Bacterial RNA extraction was performed using the Bacteria RNA Enhancement Reagent (Vazyme Biotech Co., Ltd., Nanjing, China) and VeZol reagent (Vazyme Biotech Co., Ltd.). The procedure followed the manufacturer’s instructions, and the extracted RNA was dissolved in DEPC-treated water. The purity and concentration of the RNA were verified by measuring the A260/A280 and A260/A230 ratios using a NanoDrop 2000 (Thermo Fisher Scientific, Waltham, MA, USA) and further confirmed by agarose gel electrophoresis.

The HiScript^®^ III RT SuperMix for qPCR (including gDNA wiper) from Vazyme was employed to synthesize complementary DNA (cDNA) for subsequent experiments. Real-time quantitative PCR (RT-qPCR) was conducted to assess the expression levels of cariogenic virulence factors in both treated and untreated *S. mutans*. Specifically, genes associated with caries formation in *S. mutans*, including ldh, atpD, and atpF, which are related to acid production and acid resistance, were investigated in this study. In addition, adhesion-related virulence genes (gbpB and spaP), exopolysaccharide synthesis-related genes (gtfB, gtfC, gtfD, and ftf), and two-component regulatory system-related genes (vicK and vicR) were also analyzed. The RT-qPCR reaction mixture was prepared using ChamQ Universal SYBR qPCR Master Mix (Vazyme Biotech Co., Ltd.) for a total volume of 20 μL. The nucleotide sequences of the primers for target genes and the reference gene (16S ribosomal RNA) are presented in Table 1. The 16S rRNA gene was selected as the reference gene because it has been widely used for normalization in bacterial RT-qPCR studies involving *S. mutans*. PCR amplification reactions were conducted using a LightCycler^®^ 480 II (Roche Diagnostics, Mannheim, Germany). The PCR amplification and dissociation curves were verified post-reaction, and the corresponding Ct values were logged. Data analysis was performed using three replicate samples, representing both treated and untreated *S. mutans* specimens. Dissociation curve analysis was performed after amplification to assess amplification specificity. Relative expression levels were calculated using the 2^−ΔΔCt^ method (ΔCt = Ct _target_ – Ct _16S rRNA_; ΔΔCt = ΔCt _treated_ – ΔCt _control_).

### 2.12. Cell Viability Assay

The cytotoxicity of patchouli alcohol was evaluated using the Cell Counting Kit-8 (CCK-8) assay. HaCaT, B16F10, and RAW264.7 cells in the logarithmic growth phase were seeded into 96-well plates at a density of 5 × 10^3^ cells/well and cultured in a humidified incubator at 37 °C with 5% CO_2_ for 24 h. The culture medium was then replaced with fresh medium containing different concentrations of patchouli alcohol. A solvent control group containing the same final concentration of DMSO as the treatment groups and a blank group without cells were included. After incubation for 24 h, the medium was removed and replaced with fresh medium containing 10% CCK-8 reagent. The plates were incubated in the dark for 1–2 h, and the absorbance was measured at 450 nm using a microplate reader. Cell viability was calculated using the following equation:

Cell viability (%) = [(OD_sample − OD_blank)/(OD_solvent control − OD_blank)] × 100

IC50 could not be reliably calculated because viability did not fall below 50% within the tested range.

### 2.13. Statistical Analysis

All experiments were performed with at least three independent biological replicates unless otherwise stated. Data are presented as the mean ± standard deviation (SD). Statistical analyses were performed using GraphPad Prism 8.02. Prior to parametric tests, data distribution and homogeneity of variance were assessed using the Shapiro–Wilk test and Brown–Forsythe test, respectively. For comparisons among multiple treatment groups, one-way analysis of variance (ANOVA) followed by Dunnett’s multiple-comparison test was used to compare each treatment group with the corresponding solvent control. For RT-qPCR analysis, statistical comparisons between the patchouli alcohol-treated group and the control group were performed using an unpaired two-tailed Student’s *t*-test based on ΔCt values, while relative expression levels were calculated using the 2^−ΔΔCt^ method for presentation. A value of *p* < 0.05 was considered statistically significant. For the established-biofilm assays, five independent replicates were included for each treatment group.

## 3. Results

### 3.1. Antibacterial Activity

The minimum inhibitory concentration (MIC) and minimum bactericidal concentration (MBC) values for *S. mutans* were determined to be 40 μg/mL and 80 μg/mL, respectively. In contrast, the MIC and MBC values for the positive control, chlorhexidine, were 0.625 μg/mL and 1.25 μg/mL, respectively. Furthermore, the solvent dimethyl sulfoxide (DMSO) used in this experiment exhibited no effect on the growth of *S. mutans*. At concentrations corresponding to the effective antibacterial range of patchouli alcohol, no marked cytotoxicity was observed in HaCaT, RAW264.7, or B16F10 cells, supporting acceptable cytocompatibility under the tested in vitro conditions (Figures S1–S3).

### 3.2. Growth Inhibition of S. mutans by Patchouli Alcohol

**Figure 1:**
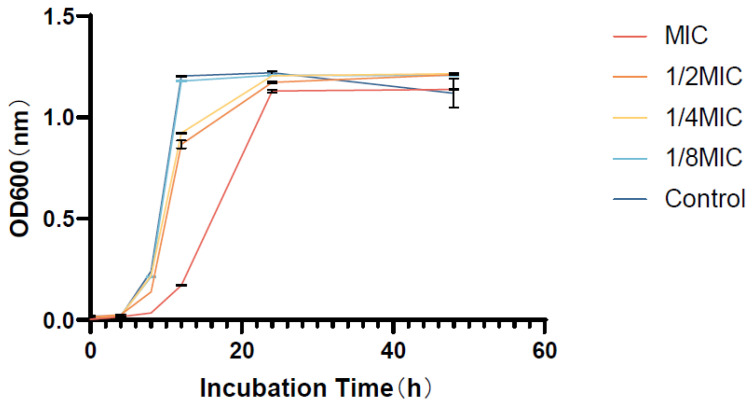
Effect of patchouli alcohol on the growth of *S. mutans*. Data are presented as the mean ± SD from three independent experiments. Statistical significance was determined at each time point using one-way ANOVA followed by Dunnett’s multiple-comparison test, comparing each treatment group with the solvent control. To avoid overcrowding the growth curve, statistical annotations were not placed directly on the plotted points. Significant differences were mainly observed at 8 h and 12 h for all tested concentrations and at 24 h for the MIC and 1/2MIC groups.

As shown in Figure 1, patchouli alcohol altered the growth profile of *S. mutans* in a concentration- and time-dependent manner. Compared with the solvent control, bacterial growth was significantly reduced at 8 h and 12 h, with the strongest inhibition observed in the MIC group. At 24 h, significant differences persisted only in the MIC and 1/2MIC groups, whereas the 1/4MIC and 1/8MIC groups were comparable to the control. By 48 h, no significant differences were detected between the treatment groups and the control. These results suggest that patchouli alcohol delayed the early-to-middle growth phase of *S. mutans*, but the inhibitory effect diminished after prolonged incubation. In the compound-only control test, OD600 values for BHI medium containing patchouli alcohol were comparable to those of blank BHI medium.

### 3.3. Inhibition of Acid Production

**Figure 2:**
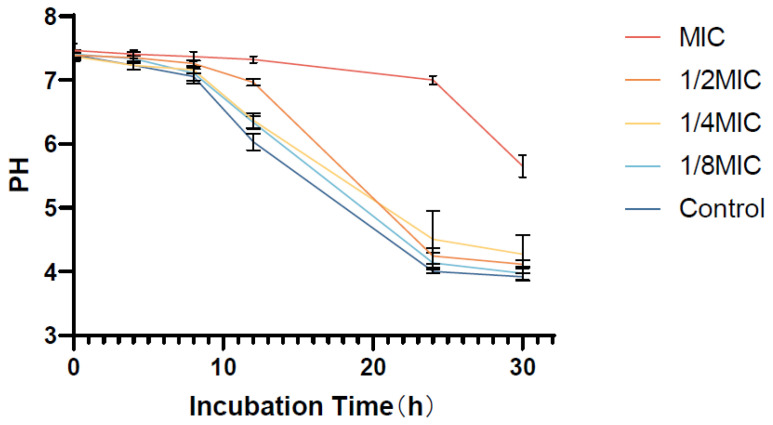
Effect of patchouli alcohol on the acidification profile of *S. mutans* cultures. The pH of the culture supernatant was measured after treatment with different concentrations of patchouli alcohol. Data are presented as the mean ± SD from three independent experiments.

Patchouli alcohol delayed the acidification of *S. mutans* cultures, particularly at the MIC concentration. At 24 h, the pH of the MIC group remained close to neutral, whereas the control and sub-MIC groups had decreased to approximately pH 4.0. At 30 h, the MIC group continued to maintain a higher pH than the control and sub-MIC groups, although acidification had progressed compared with earlier time points (Figure 2). These results indicate that patchouli alcohol primarily delayed culture acidification at the MIC concentration, whereas sub-MIC treatments produced only limited changes in pH.

### 3.4. Reduction in Established Biofilm Biomass and Metabolic Activity

**Figure 3:**
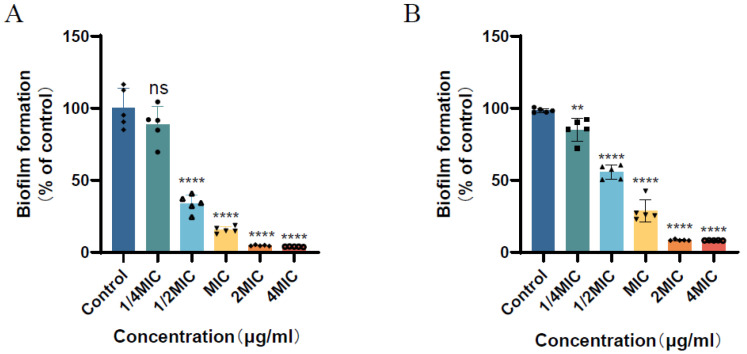
Effects of patchouli alcohol on established *S. mutans* biofilms: (**A**) Residual biofilm biomass determined by crystal violet staining. (**B**) Metabolic activity of established biofilms determined by the MTT assay. Five independent replicates were included for each treatment group; therefore, n = 5 refers to these independent replicates rather than triplicate measurements. Data are presented as the mean ± SD. Different symbol shapes were used only to distinguish the two assay panels and do not indicate different experimental groups. Statistical significance was determined using one-way ANOVA followed by Dunnett’s multiple-comparison test. ** *p* < 0.01, **** *p* < 0.0001 compared with the solvent control group; ns, not significant.

The effects of patchouli alcohol on established *S. mutans* biofilms were evaluated using crystal violet staining and the MTT assay (Figure 3). Crystal violet staining showed that residual biofilm biomass was significantly reduced in the 1/2MIC, MIC, 2MIC, and 4MIC groups compared with the control group, whereas the 1/4MIC group showed no significant difference. In contrast, the MTT assay showed a significant reduction in biofilm metabolic activity in the 1/4MIC, 1/2MIC, MIC, 2MIC, and 4MIC groups. These results suggest that patchouli alcohol reduced both the biomass and metabolic activity of established *S. mutans* biofilms. The residual established-biofilm biomass was reduced by approximately 80% at 40 μg/mL (MIC).

### 3.5. Scanning Electron Microscopy (SEM) Observations

**Figure 4:**
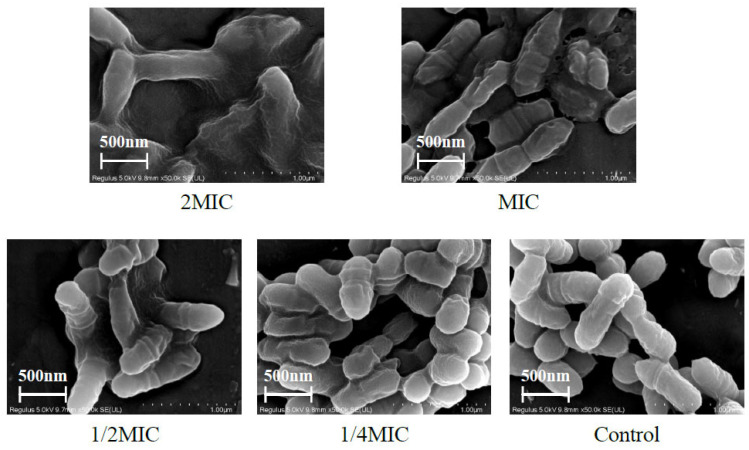
SEM observation of *S. mutans* morphology after treatment with different concentrations of patchouli alcohol. Treatment-associated surface deformation became more apparent with increasing patchouli alcohol concentration. Images were acquired at ×50,000 magnification.

Scanning electron microscopy (SEM) was employed to observe the morphological changes in *S. mutans* following treatment with patchouli alcohol. As shown in Figure 4, the surface of the bacteria displayed pronounced folds and crumples with increasing concentrations of patchouli alcohol, suggesting cell-envelope perturbation.

### 3.6. Inhibition of Exopolysaccharide Production

**Figure 5:**
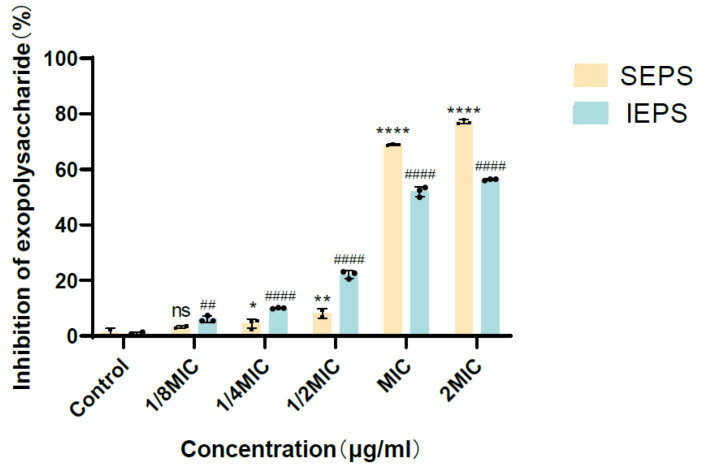
Inhibitory effect of patchouli alcohol on water-soluble exopolysaccharides (SEPS) and water-insoluble exopolysaccharides (IEPS) of *S. mutans*. Data are presented as the mean ± SD from three independent experiments. Different symbol shapes were used only to distinguish the two assay panels and do not indicate different experimental groups. Statistical significance was determined using one-way ANOVA followed by Dunnett’s multiple-comparison test. Asterisks indicate significant differences for SEPS compared with the control group, whereas hash symbols indicate significant differences for IEPS compared with the control group. * *p* < 0.05, ** *p* < 0.01, **** *p* < 0.0001; ## *p* < 0.01, #### *p* < 0.0001; ns, not significant.

*S. mutans* generates lactic acid by metabolizing sucrose and produces extracellular polysaccharides that contribute to biofilm formation. This biofilm envelops the bacteria, protecting them from salivary clearance and antimicrobial action, ultimately leading to the demineralization of dental hard tissue; thus, inhibiting exopolysaccharide production becomes a crucial target in the treatment of dental caries [16]. Figure 5 illustrates that, compared to the control group, the 1/4MIC, 1/2MIC, MIC, and 2MIC groups significantly inhibited the production of both water-soluble and water-insoluble exopolysaccharides from *S. mutans*.

### 3.7. Patchouli Alcohol Increased the Proportion of Membrane-Compromised S. mutans Cells

**Figure 6:**
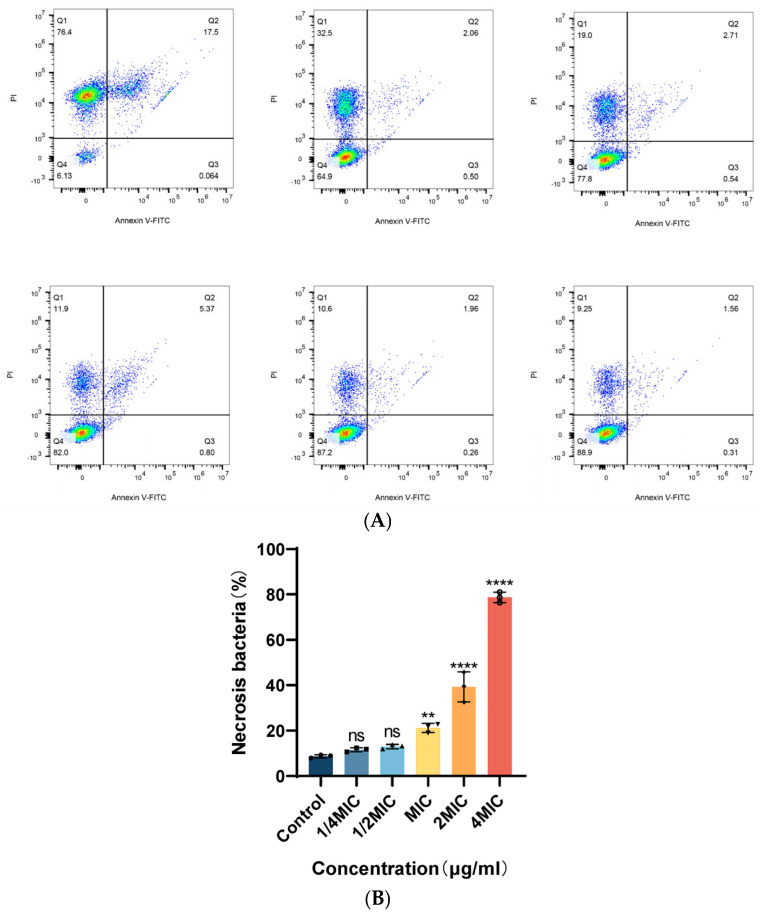
Effect of patchouli alcohol on membrane integrity of *S. mutans* as assessed by Annexin V-FITC/PI staining: (**A**) Representative flow-cytometry plots of *S. mutans* after treatment with different concentrations of patchouli alcohol. Q1 represents Annexin V−/PI+ cells, Q2 represents Annexin V+/PI+ cells, Q3 represents Annexin V+/PI− cells, and Q4 represents Annexin V−/PI− cells. These quadrants were used to describe staining patterns rather than bacterial apoptosis or necrosis. (**B**) Proportion of Annexin V−/PI+ *S. mutans* cells, interpreted as PI-positive cells with impaired membrane integrity rather than as necrotic cells. Bar colors indicate different treatment groups and patchouli alcohol concentrations, and are used only for visual distinction. Individual symbols represent independent biological replicates; different symbol shapes are used only to display individual replicate values and do not indicate different experimental conditions. Statistical significance was determined using one-way ANOVA followed by Dunnett’s multiple-comparison test. Data are presented as the mean ± SD from three independent biological replicates. ** *p* < 0.01 and **** *p* < 0.0001 compared with the solvent control group; ns, not significant.

Patchouli alcohol treatment increased the proportion of Annexin V−/PI+ *S. mutans* cells in a concentration-dependent manner, suggesting increased membrane impairment. In Figure 6, Q1 represents Annexin V−/PI+ cells, Q2 represents Annexin V+/PI+ cells, Q3 represents Annexin V+/PI− cells, and Q4 represents Annexin V−/PI− cells. These staining patterns were interpreted as changes in bacterial membrane integrity rather than as evidence of bacterial apoptosis or necrosis.

### 3.8. Effect on Expression of Virulence Genes

**Figure 7:**
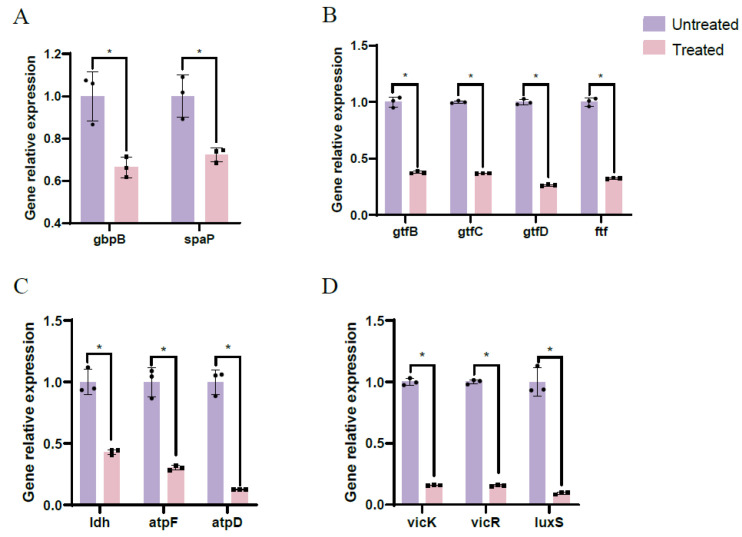
Effect of patchouli alcohol on the expression of virulence-related genes in *S. mutans*: (**A**) adhesion-related genes; (**B**) exopolysaccharide synthesis-related genes; (**C**) acid production- and acid resistance-related genes; and (**D**) two-component regulatory system- and quorum sensing-related genes. Relative expression levels were calculated using the 2^−ΔΔCt^ method and normalized to 16S rRNA. The control group was set to 1 and used as the reference baseline for relative expression analysis. Statistical significance was determined using an unpaired two-tailed Student’s *t*-test based on ΔCt values (n = 3 independent biological replicates). Different symbol shapes were used only to distinguish the two assay panels and do not indicate different experimental groups. Data are presented as the mean ± SD. * *p* < 0.05.

The expression levels of virulence factors in *S. mutans* were assessed to determine whether patchouli alcohol could significantly reduce the expression of target genes (Figure 7). Treatment with 20 μg/mL (1/2MIC) of patchouli alcohol resulted in downregulation of adhesion-related genes (gbpB and spaP), genes related to extracellular polysaccharide synthesis (gtfB, gtfC, gtfD, and ftf), and genes associated with acid production and acid resistance (ldh, atpF, and atpD). Notably, the expression levels of genes related to the two-component regulatory system and quorum sensing system (vicK, vicR, and luxS) were significantly decreased. It was determined that treatment with patchouli alcohol led to a significant reduction in the expression of virulence factors associated with adhesion, extracellular polysaccharide synthesis, acid production, and acid resistance, thereby suggesting that these transcriptional changes may be associated with reduced adhesion, acid production, EPS synthesis, and signaling activity.

## 4. Discussion

Dental caries remains one of the most prevalent oral diseases and continues to impose a substantial burden, particularly in children [17]. Natural products have been investigated as adjunctive approaches for controlling cariogenic bacteria and biofilm-associated virulence traits [18]. Among these, patchouli alcohol has shown broad-spectrum antibacterial activity against 107 Gram-positive and 20 Gram-negative strains in vitro and in vivo [19]. In the present study, patchouli alcohol delayed bacterial growth and culture acidification, reduced EPS synthesis, and decreased the biomass and metabolic activity of established *S. mutans* biofilms, with clear effects observed at the MIC concentration (40 μg/mL). In addition, sub-MIC treatment (20 μg/mL) significantly downregulated virulence-associated genes involved in adhesion (gbpB and spaP), EPS synthesis (gtfB, gtfC, gtfD, and ftf), acid production and acid tolerance (ldh, atpF, and atpD), two-component regulation (vicK and vicR), and quorum sensing (luxS).

*S. mutans* adheres to tooth surfaces through sucrose-independent and sucrose-dependent mechanisms. The former is primarily mediated by SpaP (antigen I/II), which binds components of the salivary pellicle to facilitate initial colonization [20,21] and is central to establishing the oral microecological environment [22]. GbpB, encoded by *gbpB*, anchors cells to glucan in the plaque matrix [23], enhances GTF activity, and increases cell-wall hydrophobicity and acid resistance to promote biofilm persistence [24]. The three glucosyltransferases, GtfB, GtfC, and GtfD, encoded by *gtfB*, *gtfC*, and *gtfD* [25], synthesize structurally distinct glucans from sucrose [26,27]: GtfB produces insoluble α-1,3-linked glucan, GtfC yields a mixture of soluble and insoluble glucan, and GtfD generates soluble glucan [28,29]. Fructosyltransferase (FTF, encoded by *ftf*) [30] synthesizes fructan polymers from sucrose [31]; these serve as extracellular carbohydrate reserves and, upon hydrolysis, supply glycolytic substrate for lactic acid production and enamel demineralization [32,33]. Fructans also promote bacterial adhesion to hard surfaces [34].

The acid-producing and acid-tolerant capacity of *S. mutans* is central to its cariogenicity. *S. mutans* ferments dietary sugars to produce organic acids [35]; lactate dehydrogenase (LDH, encoded by *ldh*) catalyzes the conversion of pyruvate to lactic acid in the Embden–Meyerhof pathway [36], while F1F0-ATPase (encoded by *atpD*) expels H^+^ to maintain intracellular pH homeostasis and, under acidic conditions, harnesses the proton motive force to generate ATP for bacterial growth [37,38].

The VicRK two-component system comprises the histidine kinase VicK and the response regulator VicR [39] and modulates virulence through phosphorylation-based signal transduction [40]. VicR directly regulates transcription of *gtfB* and *gtfC* [41], and the system as a whole influences acid production and acid resistance [42] as well as biofilm formation and EPS synthesis [43].

Quorum sensing coordinates biofilm development and virulence factor expression across species [44]. LuxS synthesizes autoinducer-2 (AI-2), and deletion of *luxS* markedly reduces *S. mutans* biofilm formation [45]. As a component of the activated methyl cycle, LuxS inhibition may also impair methionine metabolism and exert broader bactericidal effects [46], suggesting that quorum sensing regulation may be relevant to anti-caries strategies [47].

Dental caries is driven by ecological dysbiosis within complex multispecies oral biofilms, whereas the present study was conducted in vitro using a single reference strain of *S. mutans*. Thus, the results provide evidence from a simplified single-species model and may not fully capture the microbial complexity of the oral environment. The RT-qPCR data suggest transcriptional modulation of virulence-associated genes but do not establish corresponding changes in protein abundance or enzyme activity. Moreover, gene expression was examined at only one sub-MIC concentration; dose–response analyses and functional validation would therefore help clarify the relationship between transcriptional regulation and phenotypic inhibition. SEM observations should be interpreted with caution because dehydration and sample preparation can affect bacterial morphology; these images therefore provide supportive morphological evidence rather than direct proof of cell destruction. Although preliminary cytotoxicity assays indicated acceptable cytocompatibility under the tested conditions, further validation in oral-cell models, multispecies biofilms, three-dimensional tissue systems, and in vivo models is required before the translational potential of patchouli alcohol can be fully assessed.

## 5. Conclusions

Patchouli alcohol delayed bacterial growth and culture acidification, reduced extracellular polysaccharide synthesis, decreased established-biofilm biomass and metabolic activity, and downregulated virulence-associated gene expression in *S. mutans* under in vitro conditions. These effects were accompanied by reduced expression of genes related to adhesion, acid production, extracellular polysaccharide synthesis, two-component regulation, and quorum sensing. Together, these findings suggest that patchouli alcohol may serve as a potential natural anti-cariogenic candidate targeting *S. mutans*-associated virulence traits. Further validation in multispecies oral biofilm models, host-cell systems, and in vivo settings is required before its translational or clinical application can be considered.

## Figures and Tables

**Table 1:** Nucleotide sequences of primers used for qRT-PCR.

Gene	Primer Sequence (5′-3′)	
	Forward	Reverse
16s RNA	GAGTACGACCGCAAGGTTGA	ACCTGTCTCCGATGTACCGA
*gtfD*	ACCGTTGCTGTCGCTTCTGG	TCTGAGGTCTGCTGTTCTGTTTCTG
*lacA*	GGTGCGGGCAGCTTTATGGTAG	CGCATTATTATGGCCGCGTGTC
*lacB*	ATGTATGTGCGGTACTGGTGTGG	CCGCCAAAGCTAACGACATTTGC
*lacC*	GGTGCAGCTAAACCAACTGTCATC	GCAGCAAGAGCCGATGTAATTCC
*fsa*	TTGGTGTTGTGGACGGTGTTACC	TCCGGTGACCTCTGCACTGATAG
*galK*	TGTCTACGGCGAAGGATCTCTGC	TTCCAGGAGAGCGAGCGAAGAG

## Data Availability

The data that support the findings of this study are available from the corresponding author upon reasonable request.

## Supplementary Materials

The following supporting information can be downloaded at https://www.mdpi.com/article/10.3390/molecules31152577/s1, Figure S1. Effect of patchouli alcohol on the viability of HaCaT cells determined by the CCK-8 assay. HaCaT cells were treated with different concentrations of patchouli alcohol for 24 h, and cell viability was expressed as a percentage of the solvent control. Data are presented as the mean ± SD; Figure S2. Effect of patchouli alcohol on the viability of B16F10 cells determined by the CCK-8 assay. B16F10 cells were exposed to different concentrations of patchouli alcohol for 24 h, and cell viability was calculated relative to the solvent control. Data are presented as the mean ± SD; Figure S3. Effect of patchouli alcohol on the viability of RAW264.7 cells determined by the CCK-8 assay. RAW264.7 cells were treated with different concentrations of patchouli alcohol for 24 h, and cell viability was expressed as a percentage of the solvent control. Data are presented as the mean ± SD.
