# Supplementary material for: Patchouli Alcohol as a Potential Anti-Cariogenic Compound Targeting Streptococcus mutans
Source: Molecules. 2026 Jul 24;31(15):2577. doi: 10.3390/molecules31152577 (PMC13468659; doi:10.3390/molecules31152577)
Supplement: Supplementary file 1 [file molecules-31-02577-s001.zip › molecules-4421600-supplementary.pdf]

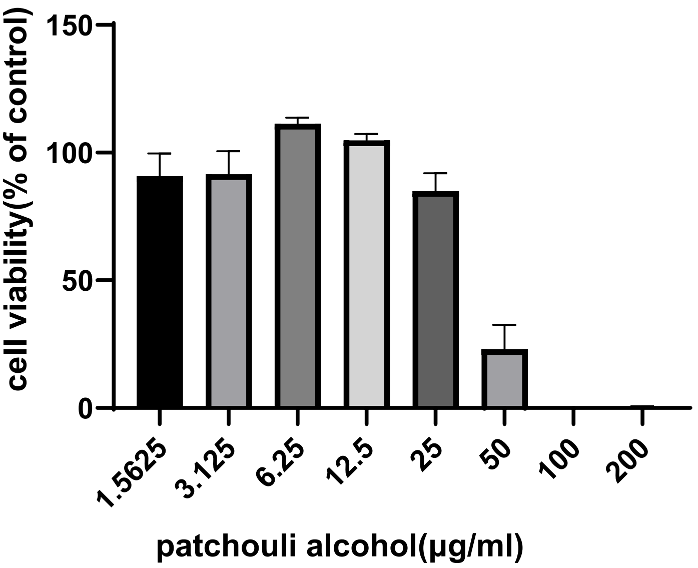

Figure S1. Effect of patchouli alcohol on the viability of HaCaT cells determined by the CCK-8 assay. HaCaT cells were treated with different concentrations of patchouli alcohol for 24 h, and cell viability was expressed as a percentage of the solvent control. Data are presented as the mean ± SD.

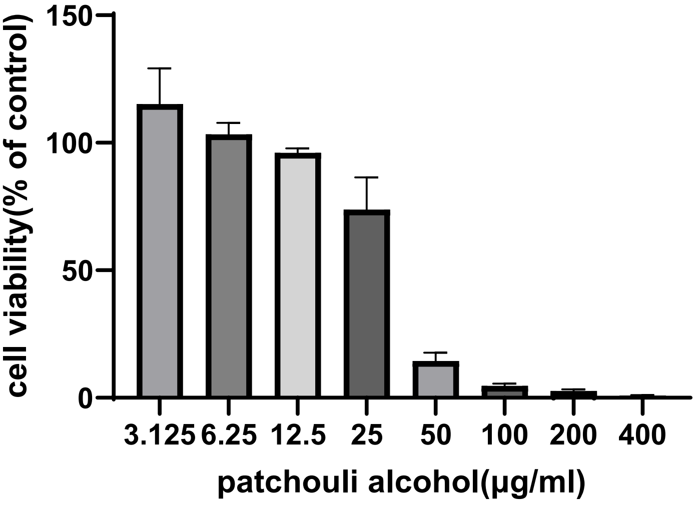

Figure S2. Effect of patchouli alcohol on the viability of B16F10 cells determined by the CCK-8 assay. B16F10 cells were exposed to different concentrations of patchouli alcohol for 24 h, and cell viability was calculated relative to the solvent control. Data are presented as the mean ± SD.

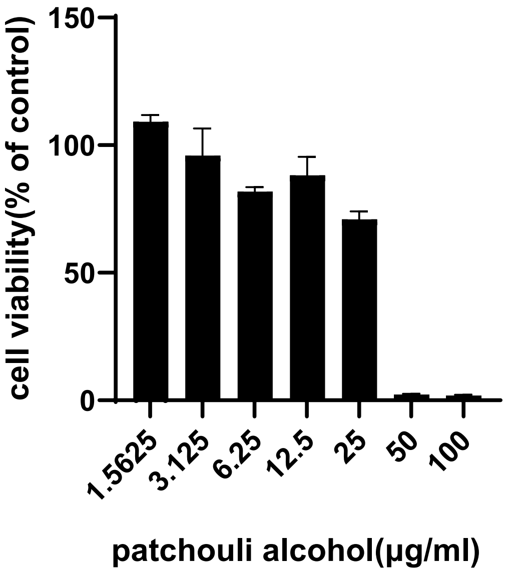

Figure S3. Effect of patchouli alcohol on the viability of RAW264.7 cells determined by the CCK-8 assay. RAW264.7 cells were treated with different concentrations of patchouli alcohol for 24 h, and cell viability was expressed as a percentage of the solvent control. Data are presented as the mean ± SD.
